# Pulmonary Aspergilloma in a Young Immunocompetent Female: A Rare Clinical Dilemma

**DOI:** 10.7759/cureus.22724

**Published:** 2022-02-28

**Authors:** Amna Rasheed, Audrey McCloskey, Shahin Foroutan, Abdul Waheed, Ariel Rodgers, Siamak M Seraj, Frederick D Cason

**Affiliations:** 1 Surgery, San Joaquin General Hospital, French Camp, USA; 2 Surgery, St. George's University School of Medicine, St. Georges, GRD; 3 General Surgery, San Joaquin General Hospital, French Camp, USA; 4 Internal Medicine, San Joaquin General Hospital, French Camp, USA

**Keywords:** young, immunocompetent, surgery, aspergilloma, pulmonary

## Abstract

Depending on the host's immunological and respiratory systems, *Aspergillus* can induce infectious and allergic diseases. Most of the spread occurs in immunocompromised people, whereas aggressive disorder in immunocompetent patients is unusual. We report the case of a 19-year-old female who had shortness of breath, right-sided chest discomfort, and intermittent hemoptysis for six months before being diagnosed with pulmonary aspergilloma. The initial chest x-ray revealed a massive right pneumothorax and a 7.2 cm rounded opacity in the right lower lung. A subsequent computed tomography (CT) chest with contrast revealed a 6.7 cm cavitating mass occupying the right lower lobe. An open right thoracotomy and right lower lobectomy showed a cavitary fungus ball with septate branching hyphae and subsequent methenamine silver staining consistent with *Aspergillus* in conjunction with a positive *Aspergillus* antigen. We strongly suggest that pulmonary aspergillosis should be suspected regardless of age or immunocompetence in patients with prolonged cough, hemoptysis, unilateral chest discomfort, and pneumothorax.

## Introduction

Pulmonary aspergilloma (PA) is rare, with a prevalence of only 18/100,000 globally, mostly affecting patients with an underlying immunocompromised state [1]. PA in individuals with no underlying immunocompromised state is extremely rare and only makes up 0.13% of all cases [2]. A subtype of chronic bronchopulmonary aspergillosis (CBA), PA is defined by the colonization of *Aspergillus* fungus within the pulmonary cavity, leading to an invasive fungal ball or mycetoma [3]. These mycetomas commonly affect immunocompromised individuals or those with compromised bronchopulmonary defenses and are frequently found secondary to an existing cavity in the lung parenchyma [2,3].

Likewise, patients suffering from PA can develop symptoms like bronchopneumonia, such as a fever that does not respond to therapy, cough, and dyspnea [4]. Hemoptysis, which is usually minor but can be severe, may also be experienced by patients, as pleuritic chest pain from vascular invasion leads to thromboses that create tiny pulmonary infarcts [4,5]. Similarly, early PA diagnosis in immunocompromised patients is challenging. Although computed tomography (CT) chest is the most common diagnostic modality, histopathological examination of thoracoscopic or open-lung biopsy tissue is the gold standard for PA diagnosis [6]. PA is identified by septate acute branching hyphae invading lung tissue and an *Aspergillus* culture from the same site. 

PA can be treated using a variety of approaches, including pharmacological and surgical treatment. Voriconazole, a relatively new antifungal medication, is the preferred treatment for PA [7]. Amphotericin B can potentially be used as an alternate therapy. A surgical resection, such as wedge resection or lobectomy, should be considered when pharmacotherapy is ineffective [7]. The current case report describes a rare PA case in a patient without underlying immunocompromised status.

## Case presentation

A 19-year-old female with a basal metabolic index (BMI) of 40 kg/m2 presented to the emergency room with sudden shortness of breath (SOB), right-sided chest pain radiating to her back when lying down, and right upper quadrant (RUQ) pain of one-day duration. The day before, she had also experienced some discomfort in her right shoulder, nausea, and one bout of vomiting. When she coughed, she mentioned she felt pain in her chest wall. She noted occasional hemoptysis ranging from bright red to dark brown for the past six months. She also supported a productive cough with yellow sputum for one year. She denied nasal congestion, sore throat, recent sick contacts, or retrosternal chest pain.

She also denied any history of trauma, tuberculosis, asthma, allergies, or prior fungal infections. The patient did have tuberculosis exposure seven years prior, but the purified protein derivative test was negative. The patient also denied any food or drug allergies. She did have multiple pets at home, including chickens, rabbits, love birds, and a dog. She did not smoke cigarettes or marijuana. On admission, vital signs were a heart rate of 120 beats per minute, respiratory rate of 22 breaths per minute, blood pressure of 104/64 mmHg, and a temperature of 37.4°C with an oxygen saturation of 99% on room air. Physical examination revealed that the right lung had diminished breath sounds with coarse crackles at the base on auscultation. Left lung breath sounds were clear to auscultation with no crackles or wheezing. 

Lab results were pertinent with a negative coronavirus disease 2019 (COVID-19) test, a white blood count of 9.6 x10 (3)/u with an absolute neutrophil count was 6.9 x10(3)/uL, lymphocyte count was 1.2 x10(3)/uL, monocyte count 0.9 x10(3)/uL, eosinophil count was 0.4 x10(3)/uL, hemoglobin was 11 gm/dL, hematocrit was 32.8 %, and platelets were 305 x10(3)/uL. Blood and urine cultures were collected, and results returned negative for any growth for five days. Also, the Initial chest x-ray demonstrated a large right pneumothorax with right lung collapse and right-sided pleural effusion. 

**Figure 1:**
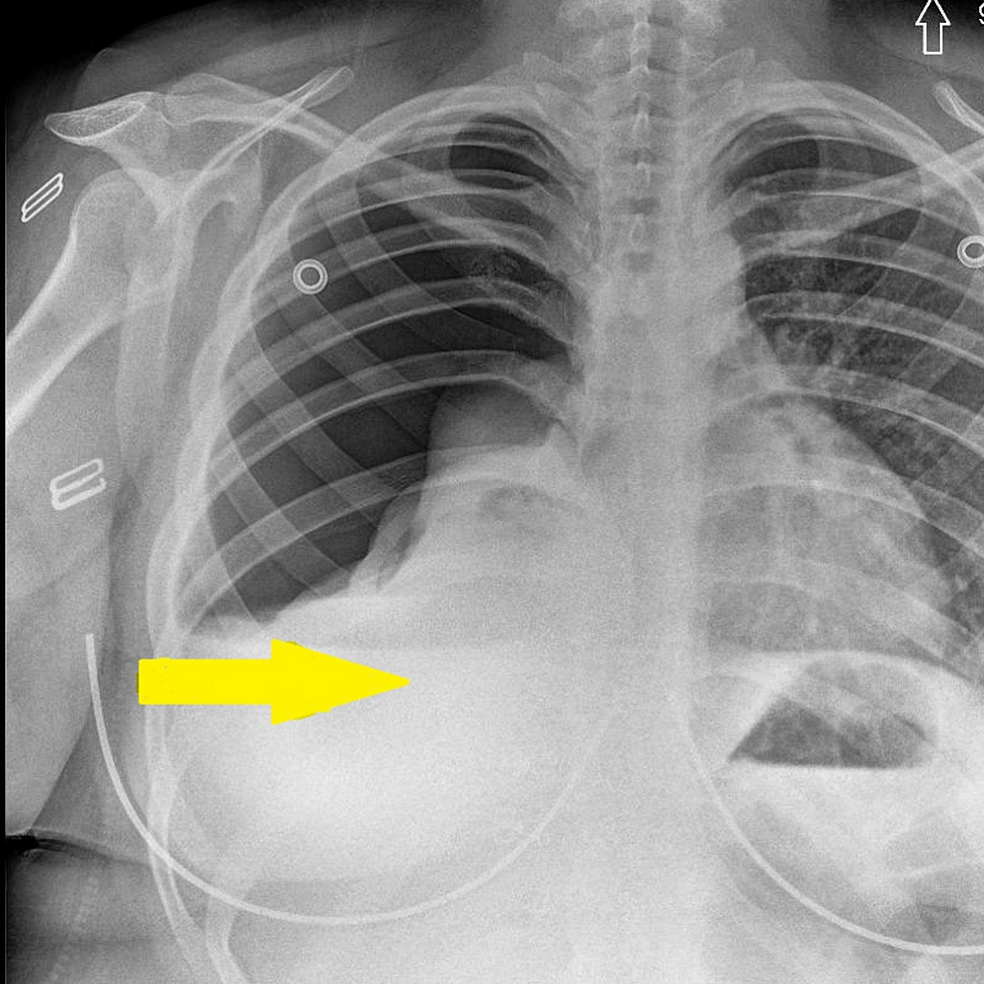
Chest x-ray (yellow arrow indicates lesion site).

A chest tube was placed in the right chest, and pneumothorax was resolved. Repeat chest x-ray showed a right chest tube with minimal right apical pneumothorax and a 7.2 cm rounded opacity in the right lower lung (Figure 1).

**Figure 2:**
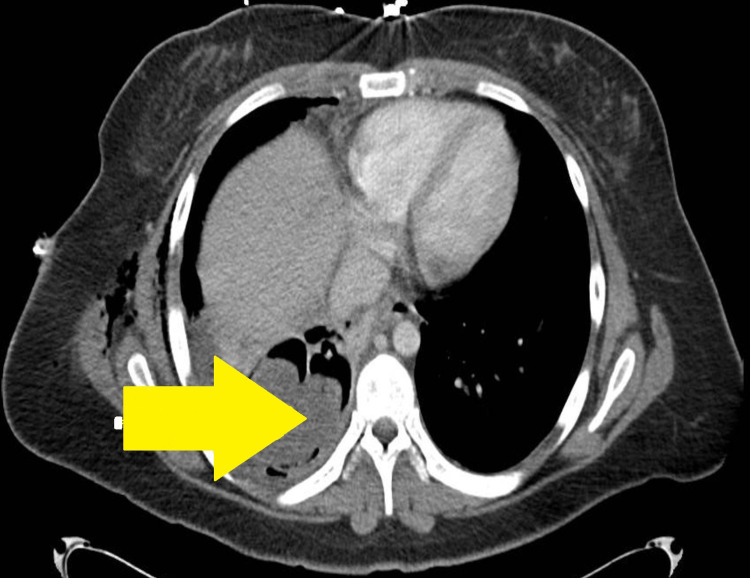
CT chest (yellow arrow indicates a lesion).

Subsequent CT chest with contrast showed a 6.7 cm cavitating mass occupying the right lower lobe and surrounding parenchymal consolidation (Figure 2).

Small right pleural effusion and trace left pleural fluid with minimal subjacent subsegmental atelectasis. No axillary, hilar or mediastinal lymphadenopathy was present. Furthermore, thoracentesis yielded red cloudy right pleural fluid with a white blood cell count of 3256 cells/mm2 consisting of 83% neutrophils, 15% lymphocytes, and 2% monocytes. The red blood cell count was 45,425 cells/mm2. Lactate dehydrogenase was 1705 IU/L, glucose was 16 mg/dL, and protein was five g/dL. Bronchoscopy showed slightly edematous mucosa with scant thick white to tan, brownish secretions, no bloody secretions, and no endobronchial lesions in the right lower lobe basilar segments. Selective washings were obtained from the right lower lobe basilar segments and the right lower lobe superior segment.

Bronchial washings showed reactive bronchial epithelium with chronic inflammatory cells, but no fungal organisms were identified for pathology. 1,3-beta-D-glucan was positive at 171 pg/mL, HIV screen was negative, antinuclear antibodies screen was negative, and antineutrophilic cytoplasmic autoantibody screen was negative. Bronchial washings for acid-fast bacillus nucleic acid amplification test were negative. *Aspergillus niger* antibodies screen and *Aspergillus fumigatus* antibody were negative. *Aspergillus* antigen screen, however, was positive. Histoplasma antibody screen was positive at <0.2, and coccidiosis’s titer screen was negative. For antibiotic coverage, IV ampicillin-sulbactam 1g every eight hours was administered for five weeks, oral amoxicillin 875 mg clavulanate (Augmentin) 125 mg, and oral vancomycin 200 mg tablet.

The patient did not show significant improvement, and a decision was made to take him to the OR. Open right thoracotomy and right lower pulmonary lobectomy were performed for medically intractable mycetoma with positive aspergillus antigen and persistent bronchopleural fistula. Thoracotomy showed soft, pink, and fully expanded right upper and middle lobes with no hilar lymphadenopathy. The lower lobe, however, contained a 10 cm spongy mass. There was also parietal pleura thickening along the lateral chest wall adjacent to the lower lobe. Surgical resection of the lower lobe showed sections of the cavitary fungus ball with septate branching hyphae, positive for methenamine silver stain, and consistent with *Aspergillus* in correlation with the positive *Aspergillus* antigen result. The cavity was lined by squamous metaplastic benign respiratory epithelium. 

The surrounding fibrous wall showed reactive fibrotic changes with no granulomatous reaction. Sections of lung tissue outside the cavity showed organizing pneumonia changes with interstitial fibrosis and lymphocytic infiltrates with reactive lymphoid follicle formation. Right apical and basilar chest tubes were placed and confirmed with x-rays. Subsequently, the patient was admitted to the floor for postoperative recovery. Apical chest tube removed on postoperative day nine. On postoperative day fifteen, the basilar chest tube was noted to have air leakage and thus was replaced with a Heimlich valve. 

## Discussion

Pneumoaspergillosis (also known as pulmonary aspergillosis) is a term used to describe a clinical sequela of pulmonary disorders caused by various species of the genus *Aspergillus* that spread through the airways [8]. An increased incidence of these fungal infections associated with considerable morbidity and mortality has been observed frequently in the recent era of modern science [1,2]. The etiology of PA involves the colonization of *Aspergillus* in a previously formed lung cavity [9]. Although previous or current mycobacterial infections are common predisposing factors to aspergillomas (30.2% of patients), other less common causes include chronic obstructive pulmonary disease, pneumothorax, previous lung cancer, pulmonary infarction, and pneumonia have been described [1,9].

The mechanism of aspergilloma formation is poorly known; however, a presumed mechanism in patients with previous lung pathology involves increased proteolytic enzyme activity after granulocyte recovery in patients recently treated with leukemia [10,11]. However, our patient was previously healthy and had no classic or proposed risk factors for lung cavitation predisposing her to infection. Besides previous lung cavities, additional risk factors that predispose patients to pulmonary aspergillosis include genetic defects in innate immunity such as toll-like receptor (TLR) 4, TLR 3, TLR 10, interleukin 15, vascular endothelial growth factor A, or plasminogen activator gene [12]. Furthermore, PA manifests itself with weight loss, productive cough, hemoptysis, shortness of breath, fever, and chest pain [3,11]. Symptoms should persist for greater than three months, and no overt immunocompromising conditions should be present [5]. Hemoptysis, in particular, presents a significant cause of morbidity and mortality and the incidence in patients with an aspergilloma ranging between 50-85% [5].

Additionally, diagnosing a PA in an immunocompetent patient might be challenging most of the time. Aspergillomas may be visualized on CT scans of the chest in a pulmonary or pleural cavity or an ectatic bronchus [13]. It is the most characteristic imaging feature of chronic pulmonary aspergillosis [13]. Radiography may show a rounded mass that usually moves within the cavity when the patient is repositioned, termed the Monod sign, and offer a classic “air crescent sign” [14]. Also, radiologically, aspergillomas can be classified into simple or chronic cavitary types [13-15]. Whereas simple aspergillomas may be thin-walled, have normal adjacent lung parenchyma, and no pleural involvement, chronic cavitary pulmonary aspergillosis may be more aggressive with more significant destruction of the lung parenchyma, ill-defined consolidation regions, and multiple cavities containing fungus balls and debris and fluid, and involve the pleura [13-15]. Besides radiological findings, the laboratory results play a criterial role in determining the particular species responsible for the illness. The most widely adopted test is a positive *Aspergillus* IgG antibody test with 86-98% sensitivity and 81-90% specificity [16]. Patients with aspergillomas should also be tested for serum *Aspergillus fumigatus* IgG [17]. Serum galactomannan and beta-D-glucan serum levels can be elevated, but they have relatively poor sensitivities, and their diagnostic value has not been well studied [17].

Moreover, the management of aspergillomas is both medical and surgical [18]. Small, simple aspergillomas can be managed with wedge resection, while larger simple aspergillomas or chronic cavitary aspergillomas may require a lobectomy or pneumonectomy [19]. Also, a cavernostomy and limited thoracoplasty are preferable for high-risk patients with extensive disease who may not tolerate anatomical resections [19]. A bronchial artery embolization may stabilize patients with significant hemoptysis before surgery [20]. Patients that undergo video-assisted thoracic surgery (VATS) have been shown to have a shorter length of stay and fewer complications than thoracotomy [11, 20].

Although surgery is considered the gold standard, nonsurgical options exist for those unable to undergo surgery [7]. Amphotericin B has a cure rate of approximately 10% but does not benefit after surgery and has a significant side-effect profile [7]. Itraconazole is the most tested antifungal agent, with two clinical trials showing cure rates more excellent than 60% with few side effects, but resistance to itraconazole makes voriconazole preferable [7]. Also, treatment efficacy focuses on both radiographic and clinical responses regarding follow-up.

## Conclusions

PA mainly affects immune-compromised individuals, yet immunocompetent patients are sporadic and less understood. Patients not suitable for medical treatment should be offered surgical resection. More patients need to be enrolled in clinical trials and retrospective national and international data registries to better understand this rare clinical entity.
